# Global genetic diversity of human apolipoproteins and effects on cardiovascular disease risk[S]

**DOI:** 10.1194/jlr.P086710

**Published:** 2018-08-03

**Authors:** Yitian Zhou, Reedik Mägi, Lili Milani, Volker M. Lauschke

**Affiliations:** Department of Physiology and Pharmacology, Section of Pharmacogenetics,* Karolinska Institutet, Stockholm, Sweden; Estonian Genome Center,† University of Tartu, Tartu, Estonia; Science for Life Laboratory, Department of Medical Sciences,§ Uppsala University, Uppsala, Sweden

**Keywords:** cholesterol, lipid traits, Alzheimer’s disease, population genetics

## Abstract

Abnormal plasma apolipoprotein levels are consistently implicated in CVD risk. Although 30% to 60% of their interindividual variability is genetic, common genetic variants explain only 10% to 20% of these differences. Rare genetic variants may be major sources of the missing heritability, yet quantitative evaluations of their contribution to phenotypic variability are lacking. Here, we analyzed whole-genome and whole-exome sequencing data from 138,632 individuals across seven major human populations to present a systematic overview of genetic apolipoprotein variability. We provide population-specific frequencies of 38 clinically important apolipoprotein alleles and identify further 6,875 genetic variants, 33% of which are novel and 98.7% of which are rare with minor allele frequencies <1%. We predicted the functional impact of rare variants and found that their relative importance differed drastically between genes and among ethnicities. Importantly, we validated the clinical relevance of multiple variants with predicted effects by leveraging association data from the CARDIoGRAM (Coronary Artery Disease Genomewide Replication and Meta-analysis) and Global Lipids Genetics consortia. Overall, we provide a consolidated overview of population-specific apolipoprotein genetics as a valuable data resource for scientists and clinicians, estimate the importance of rare genetic variants for the missing heritability of apolipoprotein-associated disease traits, and pinpoint multiple novel apolipoprotein variants with putative population-specific impacts on serum lipid levels.

CVD is a major cause of morbidity and mortality with an estimated worldwide aggregated lifetime risk of over 60% (1). Globally, 422 million patients suffer from CVD, and CVD causes more than 4 million deaths annually in Europe alone, accounting for 45% of all deaths (2, 3). Moreover, 10–18% of the number of years lost due to poor health, disability, or early death are allotted to CVD, resulting in a projected global cost of USD 47 trillion worldwide in the next 25 years (4).

Abnormal plasma lipid profiles constitute the single most important risk factor for CVD (5–7). Particularly, elevated non-HDL cholesterol levels and hypertriglyceridemia are considered as the main risk indicators (8, 9). Mechanistically, LDLs and VLDLs, as well as their remnants, can penetrate the endothelial lining of the arterial walls and be retained in the underlying intima, where they promote inflammation, as well as atherosclerotic plaque formation and progression (10).

Lipoprotein cargo, processing, and transport are tightly linked to apolipoprotein composition, and measurement of apolipoprotein plasma concentrations instead of lipid levels can serve as a simplified method for risk assessment in vascular disease (11, 12). In total, the human genome encodes 21 apolipoproteins, of which APOAs (APOA1, APOA2, APOA4, and APOA5), APOCs (APOC1, APOC2, and APOC3), APOB, and APOE have been demonstrated to play essential roles in triglyceride and cholesterol transport and metabolism (13). Furthermore, the atypical apolipoproteins APOH and APOM have been convincingly implicated in the regulation of postprandial triglyceride clearance, as well as lipid and HDL metabolism (14–16).

Seminal studies have firmly established the importance of selected SNPs in *APO* genes as important genetic risk factors for dyslipidemias as well as its comorbidities and sequelae. Prominent examples include the association between familial hypercholesterolemia [Online Mendelian Inheritance in Man (OMIM) identifier 144010]; increased risk of ischemic heart disease and genetic variants in *APOB* (17); and the association of the *APOA5* variants with hypertriglyceridemia (OMIM identifier 145750) in African-Americans, Spanish, and Caucasians (18); as well as *APOE* genotypes with circulating lipid levels and with coronary risk (19, 20). Besides constituting a risk factor for CVD, genetic variants in *APOE* are strongly linked to the risk of developing Alzheimer’s disease (OMIM identifier 104310) with the *ε4* haplotype increasing risk about 3.7-fold per copy, whereas the *ε2* allele is neuroprotective with an odds ratio (OR) of 0.5 per copy (21, 22).

Although these and other studies have provided important data on the prevalence of selected *APO* variants with clinical importance and their interethnic differences, frequencies of the majority of variants were mostly assessed in few heterogeneous populations with relatively small sample sizes. Furthermore, the genetic variability in *APO* genes beyond the interrogated selected subset of SNPs has not been systematically addressed. Importantly, the rapidly increasing extent of available next-generation sequencing (NGS) data provided by a multitude of population-scale sequencing projects allows us for the first time to comprehensively analyze and portray the landscape of genetic diversity and interethnic variability in *APO* genes across major worldwide populations.

In this study, we integrated whole-exome sequencing and whole-genome sequencing data from 138,632 individuals across seven major human populations to comprehensively profile the genetic diversity of 11 *APO* genes with the highest relevance for human lipid and cholesterol metabolism and transport. Based on these data, we provide a consolidated overview of population-specific frequencies of clinically important *APO* variants on an unprecedented scale. In addition, we analyzed the overall pattern of *APO* genetic diversity and identified 6,875 genetic variants, 2,270 of which were novel. We predict the functional impact of this genetic variability using computational predictions and by mapping variants to the domain structures of APOB and APOE and provide estimates for the functional importance of rare genetic variants and their contribution to the unexplained heritability in lipidemic phenotypes. By leveraging genome-wide association study (GWAS) data provided by the CARDIoGRAM (Coronary Artery Disease Genomewide Replication and Meta-analysis) and Global Lipids Genetics consortia, we confirm the overall accuracy of our computational variant assessments for predicting genetic associations with blood lipid traits and coronary artery disease (CAD) risk. The presented data constitute, to our knowledge, the most comprehensive overview of genetic variability in apolipoproteins published to date and provide important information to refine population-specific genotyping strategies for dyslipidemias, as well as CVD and neurological disease risk.

## METHODS

### Data sources

*APO* variants and their frequencies were derived from human sequencing data (123,136 whole-exome sequences and 15,496 whole-genome sequences) of 138,632 individuals (63,369 non-Finnish Europeans, 12,897 Finnish, 12,020 Africans, 9,435 East Asians, 15,391 South Asians, 17,210 Latinos, 5,076 Ashkenazi Jews, and 3,234 from other populations) provided by the Genome Aggregation Database (GnomAD) (23). Apolipoprotein copy number variant (CNV) data from 56,945 individuals were obtained from the Exome Aggregation Consortium repository. APOE and APOB protein domain structures were derived from Uniprot (http://www.uniprot.org) and the published literature (24, 25). The provided numbering of amino acid positions includes the signal peptide sequence. The suitability of short-read sequencing technologies for genomic profiling of human *APO* loci was determined on the basis of GC content and paralogue similarities, which were identified using Ensembl BioMart, and the fraction of inaccessible genome for each gene using the data provided by the 1000 Genomes Project (“strict mask”) in Python (supplemental Table S1) (26).

### In silico predictions

We assessed the functionality of all missense variants using five current computational functionality prediction algorithms (SIFT, Polyphen2, MutationAssessor, PROVEAN, and DANN) through ANNOVAR (27). In addition, variants were considered deleterious when they resulted in frameshifts, premature stop codons, loss of the start codons, or disruption of splice donor or acceptor sites. Predictive performance, utilized thresholds, and descriptions of the underlying assessment parameters, as well as the associated references, are provided in supplemental Table S2. We classified a variant as putatively functional if at least two methods predicted a deleterious effect. Signal peptides were analyzed using SignalP (Version 4.1) (28) and Signal-3L (Version 2.0) (29). Variants whose functionality could not be predicted by any algorithm were excluded.

### Variant and haplotype frequency analyses

Novel variants were defined relative to SNP database (dbSNP) release 135. Total numbers and aggregated frequencies of functional variants were calculated by averaging the values generated by five predictive algorithms. Representative haplotype frequencies were calculated by integrating variant frequencies with population-specific linkage information from the 1000 Genomes Project using LDLink (30). Rare and common variants were defined as variants with minor allele frequency (MAF) ≤0.01 and MAF > 0.01, respectively. The fraction of functional variability allotted to rare variants was computed as the aggregated frequency of rare functional variants divided by the total frequency of all functional variants for each gene.

### Associations with GWAS data

Genetic association data for lipid traits were obtained from the Global Lipids Genetics Consortium for 114,229 individuals; fasting lipid profiles were available for 97,248 (85.1%) of these (31). GWAS data for CVD risk was obtained from the CARDIoGRAM consortium [22,233 individuals with CAD and 64,762 controls, respectively (32). The functionality of genetic variants identified in GnomAD for which GWAS data were available was predicted as above, and effect sizes were compared between putatively deleterious and neutral variants.

## RESULTS

### Overview of the genetic variability profile in human *APO* genes

**Fig. 1.**
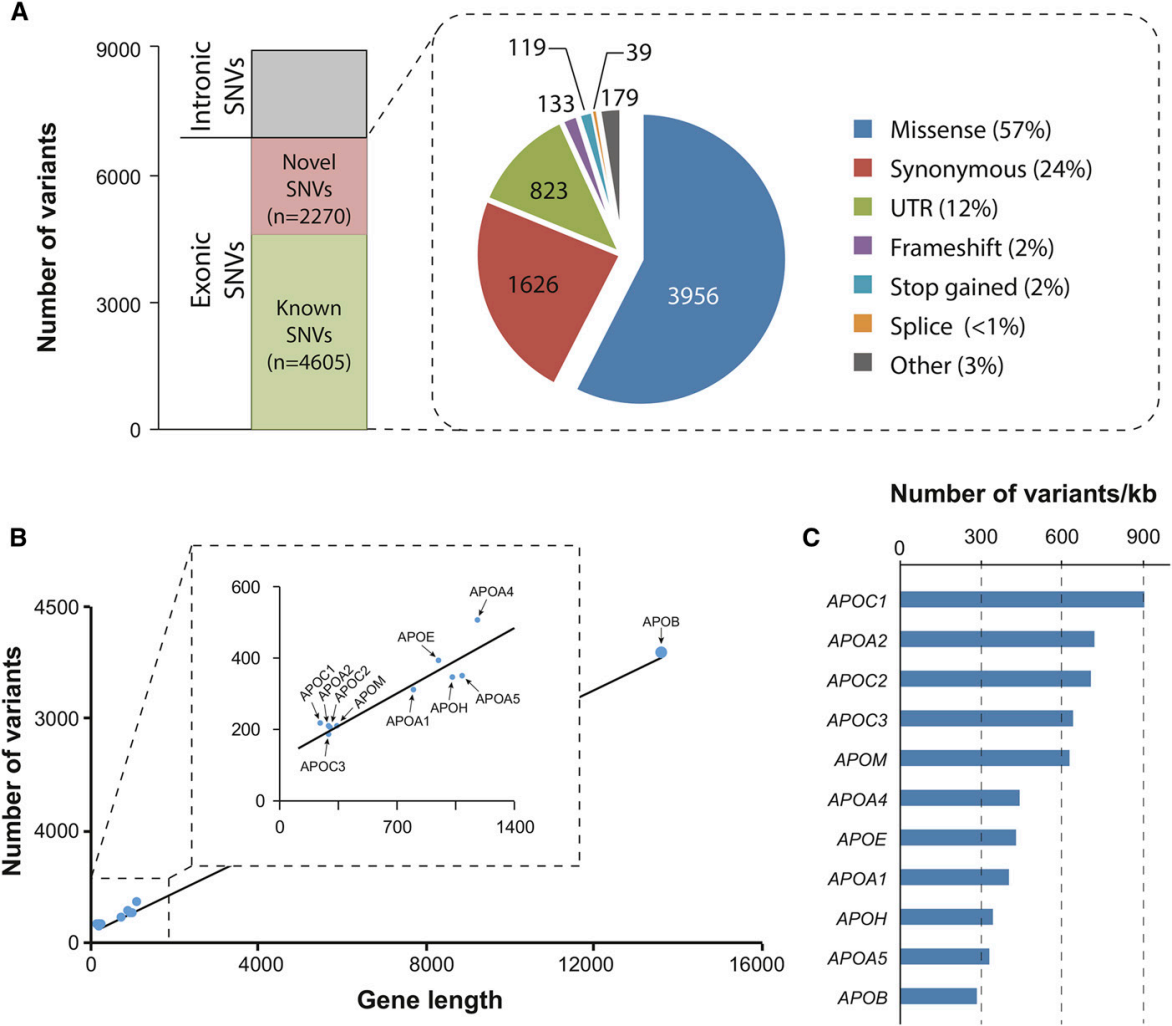
Landscape of genetic variability in human *APO* genes. A: Stacked column and pie chart showing the variant composition of the 11 analyzed *APO* genes. In total, 8,886 variants were identified in 138,632 individuals, of which 6,875 are located in exons. The majority of exonic variants are missense mutations (57%), followed by synonymous variants (24%). B: Scatter plot in which the number of variants identified in each gene is plotted against the respective gene length. Linear regression line is shown. *APOB* is by far the largest *APO* gene and harbors the most variants. C: However, when the number of variants is normalized by gene length, *APOB* was the most conserved, harboring approximately three times less variants per kilobase compared with the least conserved gene, *APOC1*.

In this study, we analyzed the genetic variability in 11 human *APO* loci with documented clinical relevance using exome and whole-genome sequencing data from 138,632 unrelated individuals from seven major human populations. In total, we identified 8,886 variants, of which 6,875 variants are located in exons (**Fig. 1A**). The majority of exonic variants are missense variants (n = 3,956; corresponding to 57% of all exonic variants), synonymous variants (n = 1,626; 24%), and variants in the untranslated region (UTR) of the mRNA (n = 823; 12%). Notably, 2,270 of these 6,875 variants are identified as novel as compared with dbSNP release 135 (Fig. 1A).

Most variants were found in the *APOB* gene (n = 3,839), followed by *APOA4* (n = 524), *APOE* (n = 408), and *APOA5* (n = 363; Fig. 1B). Yet, when analyzing the overall mutational constraints in each gene by normalizing the number of single nucleotide variants (SNVs) to the length of the corresponding transcript, we found that the gene encoding the essential apolipoprotein *APOB* was overall most highly conserved (280 SNVs per kilobase of transcript), whereas the exchangeable apolipoproteins *APOC1* (908 SNVs per kilobase), *APOA2* (717 SNVs per kilobase), and *APOC2* (706 SNVs per kilobase) showed the highest mutational burdens (Fig. 1C). Of note, however, compared with all genes in the human genome, evolutionary constraints on apolipoproteins was overall rather low (supplemental Table S3).

### Worldwide frequencies of clinically important variants in the human *APO* gene family

Genetic variants in apolipoprotein have been reproducibly linked to alterations in serum lipid profiles, progression of atherosclerosis, risk of coronary heart disease, and nonalcoholic fatty liver disease (NAFLD) (33–37). Here, we analyzed the prevalence of 38 human *APO* alleles with the highest clinical relevance in major human populations. In *APOB*, we analyzed nine SNVs (**Table 1**). The missense variant rs1042031 that was strongly associated with reduced risks of ischemic cerebrovascular disease (hazard ratio = 0.5) and stroke (hazard ratio = 0.2) in the 23-year prospective Copenhagen City Heart Study (38) differed across populations with MAFs between 4.8% in East Asians and 18.3% in Europeans. Similarly, the allele with the lead SNP rs1042034, which correlated with reduced triglyceride levels in a metaanalysis of >100,000 individuals of European ancestry (*P* < 10^−45^) (39), differed drastically in population frequencies between 27.3% in East Asians and 85.2% in African populations. In contrast, SNV rs1367117, which reproducibly correlated with increased LDL cholesterol in large metaanalyses of >180,000 individuals (*P* < 10^−196^) (40), was most and least prevalent in Europeans (MAF = 31.9%) and Africans (MAF = 11.2%), respectively. One additional *APOB* variant with strong associations with ischemic heart disease (rs5742904; OR = 7) was rare in every population analyzed (17).

**TABLE 1.** TABLE 1. Genetic diversity of a selection of clinically important *APO* variants across major human populations

		Population frequencies (in %)								
Defining variants as RSID (HGVS)	Variant type	EUR	AFR	EAS	SAS	AMR	AJ	Clinical parameters	Effect size or strength of association	Reference
*APOB*										
rs5742904 (NC_000002.11:g.21229160C>T)	Missense (R3527Q)	<0.1	<0.1	0	0	<0.1	0	Ischemic heart disease	OR = 7	(17)
rs1042031 (NC_000002.11:g.21225753C>T)	Missense (E4181K)	18.3	15.4	4.8	10.3	12.3	14.8	Ischemic cerebrovascular disease	HR = 0.5	(38)
								Ischemic stroke	HR = 0.2	
rs1367117 (NC_000002.11:g.21263900G>A)	Missense (T98I)	31.9	11.2	12.7	16	28.9	18.7	CAD	β=0.035	(40)
								LDL-C	β=0.12	
								TG	β=0.025	
								LDL	β’=4.05	(39)
rs1042034 (NC_000002.11:g.21225281C>T)	Missense (S4338N)	78.4	85.2	27.3	48.6	74.2	80.5	TG	β’=−5.99	(39)
rs693 (NC_000002.11:g.21232195G>A)	Synonymous (T2515T)	50	22.1	5.6	26.7	38	34.9	LDL-C	*P* = 7.1*10^−7^	(71)
								LDL	β=0.123	(72)
rs562338 (NC_000002.11:g.21288321A>G)	UTR	18	59.6	0.19	N.A.	16.5	29.8	LDL-C	*P* = 5.6*10^−22^	(73)
rs754523 (NC_000002.11:g.21311691A>G)	UTR	31.1	21.9	29.5	N.A.	29.1	29.1	LDL-C	*P* = 8.3*10^−12^	(73)
rs515135 (NC_000002.11:g.21286057T>C)	UTR	18.2	47.7	9.5	N.A.	18.8	30.1	CAD	OR = 1.03-1.08	(59)
rs673548 (NC_000002.11:g.21237544G>A)	Intron	22.7	21.1	73	N.A.	22	14.2	TG	β=−0.081	(72)
*APOE*										
*ε3*	Wild-type	77.4	67.5	83.6	85.7	86.4	80.6			
*ε2:* rs7412 (NC_000019.9:g.45412079C>T)	Missense (R176C)	7.7	10.8	7.5	4.2	3.2	7.8	AD	OR = 0.6-2.6	(41)
*ε4:* rs429358 (NC_000019.9:g.45411941T>C)	Missense (C130R)	14.9	21.7	8.9	10.1	10.4	11.6	AD	OR = 2.2ENTenlineENT33.1	(41)
								CAD	OR = 1.06	(19)
								NAFLD	OR = 0.51	(74)
rs4420638 (NC_000019.9:g.45422946A>G)	UTR	18.4	20.2	11.4	N.A.	10.8	14.6	LDL	β’=7.14	(39)
								LDL-C	*P* = 3.4*10^−13^	(71)
									β=0.19	(75)
rs439401 (NC_000019.9:g.45414451T>C)	UTR	36.8	15.3	58.6	N.A.	54.9	43	TG	β’=−5.5	(39)
*APOA1*										
rs670 (NC_000011.9:g.116708413C>T)	Promoter	17.3	14.8	27.8	N.A.	27.7	15.2	LDL	OR = 1.66	(76)
								TC	OR = 1.77	
*APOA2*										
rs5082 (NC_000001.10:g.161193683G>A)	Promoter	40.6	22.5	8.2	N.A.	23.4	35.8	Obesity	OR = 1.84	(77)
*APOA4*										
rs675 (NC_000011.9:g.116691675T>A)	Missense (T367S)	19.7	11.5	<0.1	12.9	9.4	21.5	CAD	HR = 2.07	(78)
rs5110 (NC_000011.9:g.116691634C>A)	Missense (Q380H)	7.8	1.5	<0.1	1.9	3.6	6.7	TG	*P* = 0.035 (+)	(79)
								VLDL	*P* = 0.035 (+)	
								HDL	*P* = 0.0005 (-)	
rs1729407 (NC_000011.9:g.116677370C>G)	Intergenic	50.8	12.7	30.4	N.A.	50.7	35.4	HDL	*P* = 7.1*10^−7^ (-)	(80)
*APOA5*										
*2: rs662799, rs651821, rs2072560, rs2266788 (NC_000011.9:g.116663707G>A, 116662579C>T, 116661826T>C, 116660686G>A),	Promoter, Kozak, Intron and UTR	8.1	0	23.8	17.7	13	N.A.	TG	20-30% elevation	(44)
*3: rs3135506 (NC_000011.9:g.116662407G>C)	Missense (S19W)	6.4	6.2	<0.1	3.8	15.3	6.8	TG	OR = 7.79	(18, 81)
rs662799 (NC_000011.9:g.116663707G>A)	Promoter	6.9	12.1	29.2	N.A.	15	10.6	TG	*P* < 0.001 (+)	(82)
								TG	*P* = 0.001 (+)	(83)
								HDL	*P* = 0.008 (-)	
rs2266788 (NC_000011.9:g.116660686G>A)	UTR	7.4	1.6	21	N.A.	13.6	10.9	TG	*P* = 33*10^−5^	(71)
								CAD	OR = 1.15	(84)
rs2075291 (NC_000011.9:g.116661392C>A)	Missense (G185C)	<0.1	0.3	6.9	0.8	<0.1	0	TG	OR = 11.73	(45)
								CAD	OR = 2.09	(85)
*APOC1*										
rs11568822 (NC_000019.9:g.45417640_45417641insCGTT)	Promoter	23*^a^*	35*^b^*	N.A.	N.A.	16.5*^c^*	N.A.	AD	OR = 1.84	(86)
*APOC3*										
rs5128 NC_000011.9:g.116703640G>C	UTR	9.5	15.5	30.3	14	CAD	OR = 1.3	(87)
rs2854116 NC_000011.9:g.116700169C>T	Promoter	39.1	70.7	41.6	47	Metabolic syndrome	OR = 1.73	(88)
				CAD	OR = 1.28	(87)
				NAFLD	*P* < 0.001 (+)	(89)
rs2854117 NC_000011.9:g.116700142T>C	Promoter	28.9	68.3	42.4	35.4	TG	*P* = 0.041 (+)	(83)
				HDL	*P* = 0.005 (-)			
				NAFLD	*P* < 0.001 (+)	(89)
rs147210663 NC_000011.9:g.116701560G>A	Missense (A43T)	<0.1	0.2	<0.1	1.1	TG	*P* = 0.01 (-)	(47)
				HDL	*P* = 0.004 (+)			
rs76353203 NC_000011.9:g.116701353C>G	Stop-gain (R19X)	<0.1	<0.1	<0.1	0	CAC	OR = 0.35	(46)
				CHD	HR = 0.68			
rs138326449 NC_000011.9:g.116701354G>A	Splice donor	0.2	<0.1	0	0.2	TG	*P* = 8*10^−8^ (-)	(90)
*APOH*										
rs8178847 NC_000017.10:g.64216815C>T	Missense (R154H)	6.7	9.8	6.3	VT	OR = 1.55	(91)
rs1801689 NC_000017.10:g.64210580A>C	Missense (C325G)	3.3	0.5	<0.1	LDL	*P* = 1* 10^−11^ (+)	(31)
rs1801690 NC_000017.10:g.64208285C>G	Missense (W335S)	5.6	0.8	6.8	TG	*P* = 0.018 (+)	(92)
				APOA-I levels	*P* = 0.026 (-)		
rs3760291 NC_000017.10:g.64226197G>T	Promoter	26.1	7.2	6.3	TC	*P* = 0.006 (-)	(14)
				LDL	*P* = 0.03 (-)		
				APOB levels	*P* = 0.001 (-)		
				APOE levels	*P* = 0.006 (-)		
*APOM*										
rs707922 NC_000006.11:g.31625507G>T	Missense (G111V)	5.2	31.9	15.9	TC	*P* = 0.006 (+)	(93)
				LDL	*P* = 0.009 (+)		
rs805296 NC_000006.11:g.31622893T>C	Promoter	1.3	11.5	11.6	CAD	OR = 1.9	(94)
				T2DM	OR = 2.29	(95)
rs940494 NC_000007.13:g.56348924A>G	Promoter	22.4	14.1	9.4	CAD	OR = 1.82	(96)

AD, Alzheimer’s disease; AFR, Africans; AJ, Ashkenazi Jews; AMR, Latinos; CAC, coronary artery calcification; CHD, coronary heart disease; EAS, East Asians; EUR, Europeans; HDL, HDL levels; HGVS, Human Genome Variation Society; HR, hazard ratio; LDL, LDL levels; LDL-C, LDL cholesterol; N.A., not available; RA, rheumatoid arthritis; RSID, reference SNP cluster ID; SAS, South Asians; T2DM, T2D mellitus; TC, total cholesterol levels; TG, triglyceride levels; VLDL, VLDL levels; VT, venous thrombosis; β, standardized effect size; β′, nonstandardized effect size.

a Obtained from Gayà-Vidal et al. (97).

b Obtained from Gao et al. (98).

c Obtained from Lucatelli et al. (99).

Genetic variability in the *APOE* gene has been reproducibly associated with differences in LDL cholesterol (19). Furthermore, *APOE* polymorphisms are the strongest risk factor for Alzheimer’s disease across genders and ethnicities with ORs for homozygous carriers of the *ε4* allele between 2.2 in Latinos and 33.1 in East Asian populations (41, 42). In agreement with previous reports (43), we found the highest frequencies of the *ε2* and *ε4* alleles in African populations, with a frequency of 10.8% and 21.7%, respectively (Table 1). In contrast, the *ε2* allele was least prevalent in Latino populations (frequency = 3.2%), whereas the frequency of the *ε4* allele was lowest in East Asians (frequency = 8.9%).

In addition, we analyzed the frequencies of 10 variant alleles in *APOA1*, *APOA2*, *APOA4*, and *APOA5* that have been consistently linked to hypertriglyceridemia and elevated risks of CAD (Table 1). APOA5*2 and APOA5*3 are both independently associated with high plasma triglyceride levels (18, 44). Importantly, whereas APOA5*2 was highly prevalent in East and South Asians (allele frequency = 23.8% and 17.7%, respectively), the allele was absent in Africans. In contrast, APOA5*3 was common in Europeans, Africans, Latinos, Ashkenazim, and South Asians with allele frequencies between 3.8% and 15.3%, but the variant was very rare in East Asian populations. The missense variant rs2075291 that strongly increases the likelihood of developing hypertriglyceridemia (OR = 11.7) (45) was only common in East Asians (MAF = 6.9%), whereas it was rare or absent in all other populations studied.

The loss-of-function mutations rs147210663, rs76353203, and rs138326449 in *APOC3* have been robustly linked to favorable lipid profiles and reduced risks of CVD (46, 47). rs147210663 has been reported to have particularly strong effects on serum triglyceride levels in Pima Indians, lowering triglyceride levels by 42% (48). In our data set, the frequency of this variant was highest in Ashkenazi Jews (MAF = 1.1%) and rare in all other populations, including Latinos (MAF < 0.1%). Similarly, the stop-gain variant rs76353203 and the splice mutation rs138326449 were rare or very rare across all populations (Table 1).

Missense and promoter SNVs in the genes encoding the atypical apolipoproteins APOH and APOM showed drastic interpopulation differences (Table 1). Prevalence of the *APOH* variant rs1801689, which was strongly associated with increased serum LDL (31), was highest in Ashkenazi Jews (MAF = 5.8%) but rare in both African (MAF = 0.5%) and East Asian (MAF < 0.1%) populations. Conversely, rs805296 located in the *APOM* promoter was most abundant in Africans (MAF = 11.5%) and East Asians (MAF = 11.6%), but less prevalent in Europeans (MAF = 1.3%) and Ashkenazim (MAF = 1%). Combined, the presented data reveal the extent of interpopulation differences in apolipoprotein alleles with demonstrated clinical relevance and provide a powerful resource for researchers and clinicians to design population-specific genotyping strategies for biomarker identification and disease-risk analyses.

### Rare genetic variants are predicted to contribute substantially to the functional variability in human apolipoproteins

**Fig. 2.**
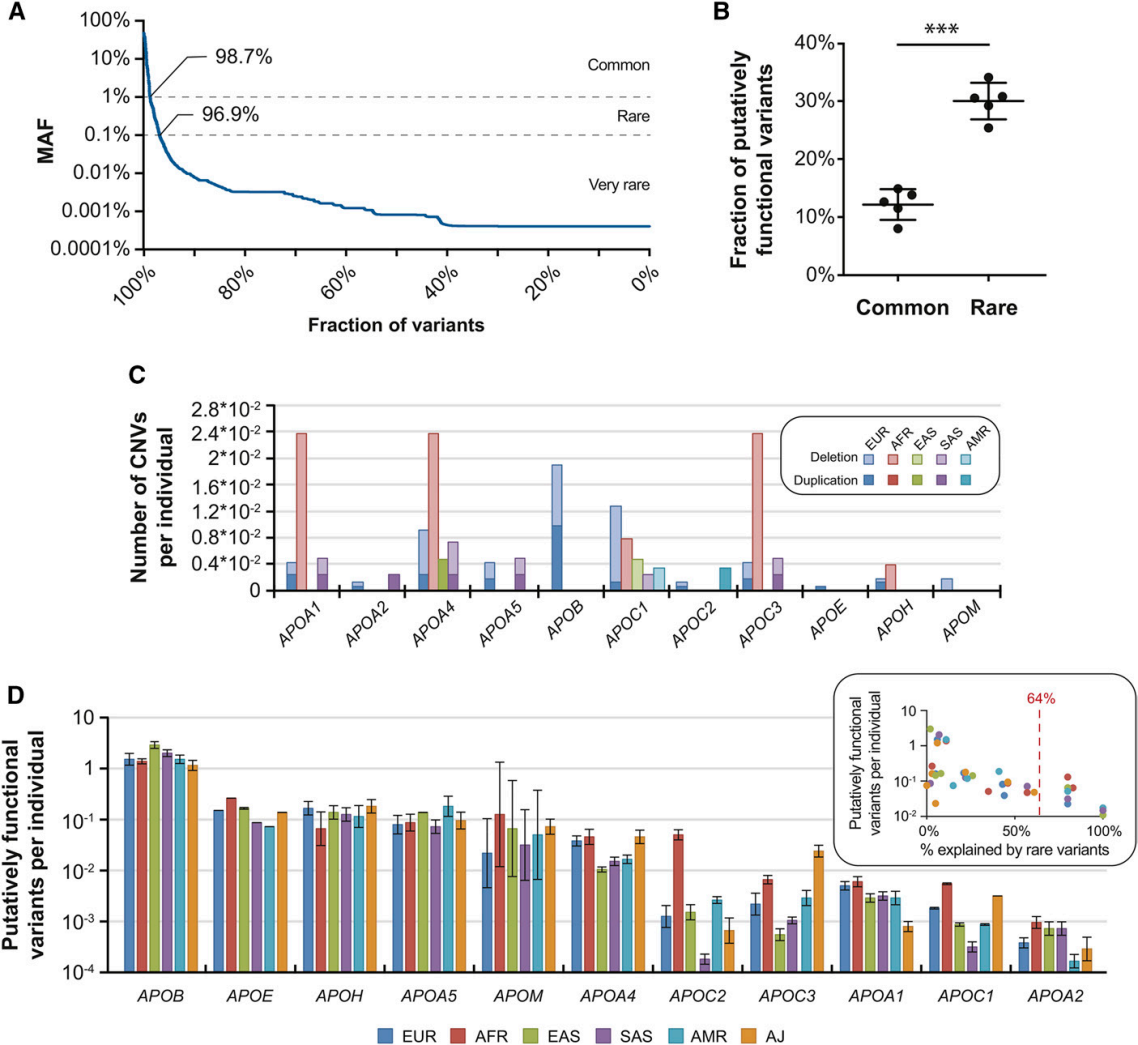
Rare genetic variants are important contributors to the functional variability of apolipoproteins. A: The vast majority of the identified apolipoprotein variants were rare (98.7%) or very rare (96.7%) with MAF < 1% or < 0.1%, respectively. B: The fraction of common and rare *APO* variants with putatively functional effects, as predicted by five computational algorithms. Note that all algorithms indicate that rare variants are enriched in mutations with functional effects. *** *P* < 0.0001 in paired heteroscedastic *t*-test. Error bars indicate SD. C: The number of CNVs per individual in *APO* genes is shown for six major human populations. AFR, Africans; AMR, Latinos; EAS, East Asians; EUR, Europeans; SAS, South Asians. Deletions and duplications are indicated in light and dark shades, respectively. Note that *APOA1*, *APOA4*, and *APOC3* are in the same locus. D: The number of variants with putatively functional consequences per individual is shown for each analyzed *APO* gene across six populations. The inset indicates the fractions of putatively functional variants that are explained by rare variants (see also supplemental Fig. S1). Average value is indicated by red dashed line. Genes with an aggregated functional variant frequency is <1% are by definition explained exclusively by rare variants and are not shown.

Importantly, the vast majority of identified exonic variants were rare (98.7%) or very rare (96.9%) with MAFs ≤1% or ≤0.1%, respectively, highlighting the genetic complexity of human *APO* genes (**Fig. 2A**). To estimate the contribution of rare genetic variants to the lipid trait variability, we predicted the overall genetically encoded functional variation in human apolipoproteins using population-scale NGS data and compared the impacts of common and rare *APO* variants. To this end, we used five functionality prediction algorithms and ensemble scores (SIFT, Polyphen2, MutationAssessor, PROVEAN, and DANN) that predict the functional impact of a variant based on a diverse set of features, including amino acid properties, secondary structure, and evolutionary conservation (supplemental Table 2).

Based on these algorithms, we detected overall 1,829 different putatively functional SNVs across human *APO* genes (27% of all exonic variants), and rare variants were significantly enriched in mutations with deleterious effects (30% of all exonic rare variants were predicted to be deleterious compared with 12.2% of common variants; Fig. 2B and supplemental Table S4). Additionally, we found novel rare CNVs in all *APO* genes studied, many of which were population-specific (Fig. 2C and supplemental Table S4). Deletions of the *APOA1*/*APOA4*/*APOC3* locus in Africans (frequency = 0.11%) and CNVs of the *APOB* gene in Europeans (0.09%) were overall most common. In contrast, very few CNVs were observed in *APOA2*, *APOM*, *APOE*, and *APOC2*.

When combining information of putatively functional SNVs and CNVs, we observed that the frequency of genetically encoded functional variability differed by more than 4,000-fold between *APO* genes. Of all analyzed *APO* genes, *APOB* harbored the most variants, with predicted functional impacts (1.8 functional variants per individual), whereas *APOA2* was highly invariant (5.4 × 10^−4^ functional variants per individual; Fig. 2D). In addition to these drastic differences in functional variability between genes, we observed large variability across populations. For *APOC2*, the genetically encoded functional variability differed 280-fold between 1.8 × 10^−4^ variants per individual in South Asians and 0.051 in Africans. In contrast, functional variability in *APOA5* (0.071–0.19 per individual), *APOB* (1.2–3 per individual), and *APOH* (0.065–0.18 per individual) differed less than 3-fold across populations (Fig. 2D).

Based on our predictions, we estimate that rare genetic variability accounts for 8% and 10% of the genetically encoded functional variability in *APOE* and *APOB*, respectively (supplemental Fig. S1). In contrast, the functional variability of *APOA4*, *APOM*, *APOA1*, *APOA2*, and members of the *APOC* gene family was fully allotted to rare variants. On average, across *APO* genes and populations, we predict that an average of 64% of the functional variability in coding sequences was due to rare genetic variants (Fig. 2D, inset). Combined, these results suggest that a substantial fraction of the genetically encoded functional variability in apolipoproteins is missed when only considering common genetic variants, thus incentivizing the consideration of rare SNVs in apolipoprotein-encoding genes for the guidance of personalized disease-risk predictions.

### Structural variability profiles of APOB and APOE

**Fig. 3.**
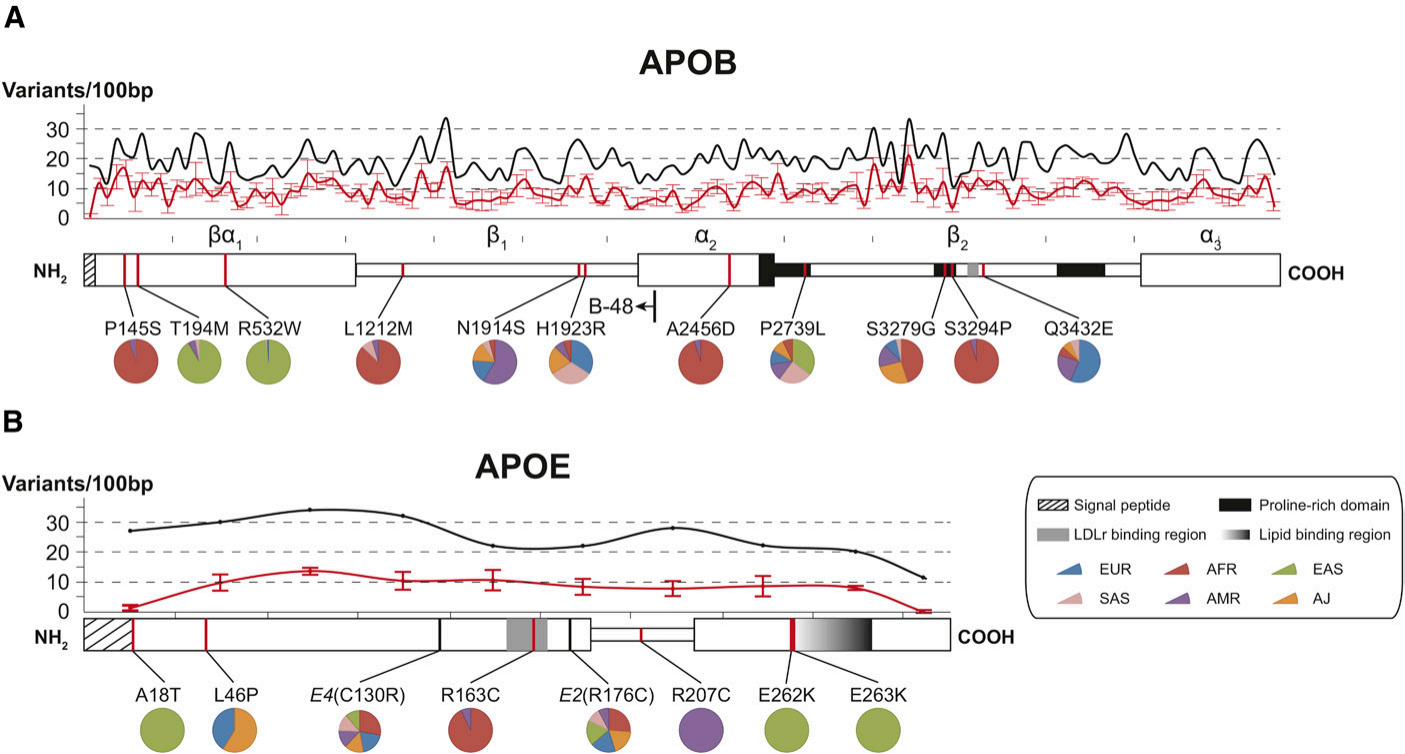
Structural variability of *APOB* and *APOE*. A: Domain map of *APOB* containing signal peptide (amino acids 1–27), proline-rich domains (amino acids 2,578–2,767; 3,243–3,318; and 3,714–3,892) (see Ref. 99), and the LDLR binding region (amino acids 3,386–3,396) (see Ref. 25). Additionally, the C terminus of the truncated APOB48 isoform is indicated. B: The domain map of *APOE* is shown with signal peptide (amino acids 1–18), LDLR binding region (amino acids 152–168), and lipid-binding region (amino acids 262–290) highlighted (see Ref. 100). Aligned line plots indicate the total number of variants (black line) and number of functional variants (red line) per 100 bp. Functionality is predicted by five orthogonal computational algorithms, and the average ± SD is shown. Variants that were common (MAF > 1%) in at least one population and deemed functional by all employed algorithms are highlighted. Pie charts indicate their relative abundance in the six major populations analyzed. AFR, Africans; AJ, Ashkenazi Jews; AMR, Latinos; EAS, East Asians; EUR, Europeans; SAS, South Asians.

Next, we mapped the genetic variability in *APOB* and *APOE* onto the respective protein domain structures (**Fig. 3**). With the exception of the N-terminal sequence encoding the signal peptide, genetic variability in *APOB* fluctuates between 11 and 34 variants per 100 bp, whereas the numbers of putatively deleterious variants range from 3.4 to 21.4 variants per 100 bp (Fig. 3A). The functionally most conserved regions are located in the amphipathic lipid-associating α-helix α_2_ and in the proline-rich domains of the β_2_ β-sheet that assumes a confirmation parallel to the phospholipid monolayer of LDL (49), with 3.4 and 3.6 variants per 100 bp, respectively. Notably, whereas the proline-rich domains in β_2_ are highly conserved (3.6 variants per 100 bp), substantially more variants were found in the adjacent LDL receptor (LDLR) binding site (9.4–13.6 variants per 100 bp). Regions of overall highest putatively functional variability are located in β-sheets β_1_ and β_2_ (17.4 and 21.4 variants per 100 bp, respectively).

To pinpoint novel variants with putative functional consequences and clinical relevance, we filtered variants that were classified as deleterious with high confidence by all algorithms employed and that were common with MAF > 1% in at least one population studied (**Table 2**). The population-specific variants rs6752026, rs13306198, and rs13306194 are located in the N-terminal αβ_1_ domain that forms a lipid pocket that is necessary for VLDL as well as chylomicron particle assembly (50). Variant rs676210, which has been previously implicated in differential lipid-lowering response to fenofibrate in Europeans (51), mapped to the proline-rich domain at the interface between the α_2_ and β_2_ regions and was the most prevalent of the putatively functional *APOB* variants with population frequencies ranging between 14.7% in Africans and 72.5% in East Asians.

**TABLE 2.** Selection of putatively functional variants in *APOB* and *APOE*

		Population Frequencies (%)						Global Lipid Genetics Consortium		CARDIoGRAM	
Defining Variants as RSID (HGVS)	Variant Type	EUR	AFR	EAS	SAS	AMR	AJ	Effect Size	*P*	Log_odds	*P*
*APOB*											
rs6752026 (NC_000002.11:g.21260934G>A)	Missense (P145S)	<0.1	12.8	0	<0.1	0.7	<0.1	N.A.	N.A.	N.A.	N.A.
rs13306198 (NC_000002.11:g.21260084G>A)	Missense (T194M)	<0.1	<0.1	5.5	0.2	0.3	0	N.A.	N.A.	N.A.	N.A.
rs13306194 (NC_000002.11:g.21252534G>A)	Missense (R532W)	0.1	<0.1	13.4	0.2	<0.1	<0.1	N.A.	N.A.	N.A.	N.A.
rs61736761 (NC_000002.11:g.21238007G>T)	Missense (L1212M)	<0.1	8.9	0	0.9	0.5	<0.1	N.A.	N.A.	N.A.	N.A.
rs1801699 (NC_000002.11:g.21233999T>C)	Missense (N1914S)	1.9	0.5	<0.1	0.6	6.5	1.6	0.091	2.6 × 10^−6^	N.A.	N.A.
rs533617 (NC_000002.11:g.21233972T>C)	Missense (H1923R)	4	0.7	<0.1	3.7	0.9	2.4	0.14	9.6 × 10^−45^	−0.096	0.034
rs12713675 (NC_000002.11:g.21232373G>T)	Missense (A2456D)	<0.1	5.3	0	<0.1	0.3	0	N.A.	N.A.	N.A.	N.A.
rs676210 (NC_000002.11:g.21231524G>A)	Missense (P2739L)	21.6	14.7	72.5	50.2	25.7	19.4	0.059	4.1 × 10^−39^	0.03	0.077
rs12720854 (NC_000002.11:g.21229905T>C)	Missense (S3279G)	0.3	1.7	<0.1	0.1	0.6	1	N.A.	N.A.	N.A.	N.A.
rs12720855 (NC_000002.11:g.21229860A>G)	Missense (S3294P)	<0.1	5.3	0	<0.1	0.3	0	N.A.	N.A.	N.A.	N.A.
rs1042023 (NC_000002.11:g.21229446G>C)	Missense (Q3432E)	1.1	0.1	0	0.1	0.5	0.1	N.A.	N.A.	N.A.	N.A.
*APOE*											
rs533904656 (NC_000019.9:g.45411025G>A)	Missense (A18T)	0	0	0.2	0	0	0	N.A.	N.A.	N.A.	N.A.
rs769452 (NC_000019.9:g.45411110T>C)	Missense (L46P)	0.3	<0.1	0	<0.1	<0.1	0.5	N.A.	N.A.	N.A.	N.A.
rs769455 (NC_000019.9:g.45412040C>T)	Missense (R163C)	<0.1	2	0	<0.1	0.2	0	N.A.	N.A.	N.A.	N.A.
rs749750245 (NC_000019.9:g.45412172C>T)	Missense (R207C)	0	0	0	0	0.2	0	N.A.	N.A.	N.A.	N.A.
rs140808909 (NC_000019.9:g.45412337G>A)	Missense (E262K)	0	0	0.3	0	0	0	N.A.	N.A.	N.A.	N.A.
rs190853081 (NC_000019.9:g.45412340G>A)	Missense (E263K)	0	0	0.3	0	0	0	N.A.	N.A.	N.A.	N.A.

AFR, Africans; AJ, Ashkenazi Jews; AMR, Latinos; EAS, East Asians; EUR, Europeans; HGVS, Human Genome Variation Society; N.A., not available; RSID, reference SNP cluster ID; SAS, South Asians.

Familial hypobetalipoproteinemia type 1 (FHBL1; OMIM identifier 615558) is caused by genetic variants that result in truncated forms of APOB protein. Heterozygous carriers of such variants (1:500 to 1:1,000 in Western populations) are often clinically asymptomatic, whereas individuals homozygous for APOB truncating mutations often exhibit very low LDL levels, fat malabsorption in the intestine, hepatic steatosis due to impaired VLDL secretion by the liver, and high prevalence of severe fibrosis (52–54). Additionally, if the truncating variant occurs within the APOB48 isoform, chylomicron secretion from the enterocytes is affected. In the 138,632 individuals analyzed here, we found only very few carriers of *APOB* truncating variants (supplemental Table S5). However, four *APOB* missense variants recently associated with FHBL1 were identified with frequencies between 0.8% and 2.5% in Africans (55) (supplemental Table S5).

In contrast to APOB, variability in APOE was distributed uniformly across the open-reading frame sequence with a local minimum at the LDLR binding region (Fig. 3B). In-depth computational analysis of the *APOE* variant inventory revealed multiple rare SNVs with high-confidence functional consequences. Variants rs533904656 (A18T), rs140808909 (E262K), and rs190853081 (E263K) were specific to East Asian populations with MAFs between 0.2% and 0.3%. The A18T alters the signal peptide sequence and obscures cleavage-site recognition (28, 29), potentially modulating APOE secretory efficiency. E262 and E263 are located in the CT domain, and the latter forms salt bridges with R121 and R165 in the LDLR-binding region, which shields the LDLR-binding domain in the absence of bound lipids (24). Thus, we hypothesize that SNVs altering the charge of this residue entail destabilization and favor premature binding of the lipid-free APOE to its receptor. A similar weakening of domain interactions can be expected for the R163C mutation that is found in 2% of African alleles and which abolishes interactions with Q59 in the NT domain (24).

### Validation of predicted associations using GWAS data

**Fig. 4.**
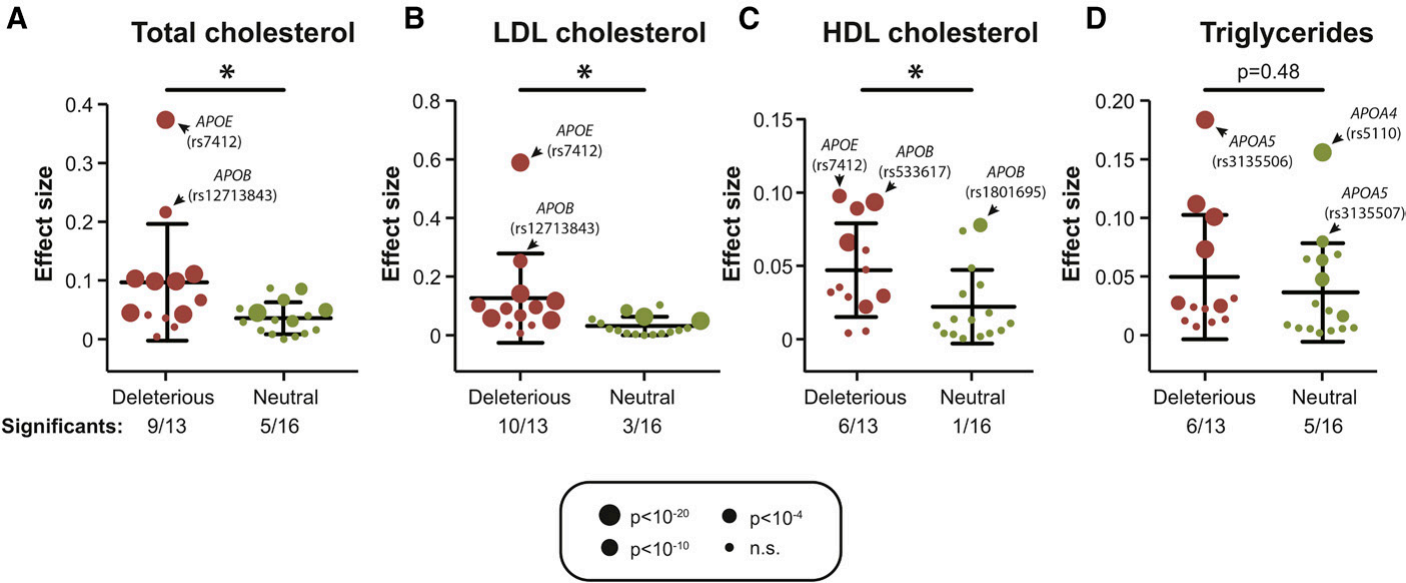
Putatively deleterious variants are enriched in mutations with effects on blood lipid traits. Identified *APO* variants were overlaid with GWAS data provided by the Global Lipids Genetics Consortium for total cholesterol (A), LDL cholesterol (B), HDL cholesterol (C), and serum triglycerides (D). Sizes of dots indicate *P* values of the associations between variant and the respective clinical parameter. *P* < 10^−4^ indicates significance of association after Bonferroni correction. Importantly, variants predicted to be deleterious (indicated in red) were significantly enriched in mutations affecting lipid traits (*P* < 0.001; chi-squared test) compared with variants predicted to be functionally neutral (indicated in green). When individual lipid parameters were compared, variant associations were significant for cholesterol traits (total, LDL, and HDL cholesterol; *P* < 0.05) but not serum triglyceride levels (*P* = 0.48; heteroscedastic two-tailed *t*-test). * *P* < 0.05; n.s., not significant.

We aimed to estimate the accuracy of these predictions by leveraging preexisting genotyping data from 114,229 individuals provided by the Global Lipids Genetics consortium (31). Of the 6,875 exonic apolipoprotein variants identified in this study, only 51 overlapped with GWAS data (0.7%; supplemental Table S6). This demonstrates the vast extent of genetic complexity not interrogated by genome-wide and custom genotyping arrays and emphasizes the added value of sequencing-based profiling techniques. Importantly, we found that apolipoprotein variants that were predicted to affect the functionality of the corresponding gene product showed significantly higher effect sizes for cholesterol traits, including total, LDL, and HDL cholesterol (*P* < 0.05 for each correlation; **Fig. 4A**–C). Of variants with putatively deleterious effects, 69% (9/13), 77% (10/13), and 46% (6/13) significantly correlated with changes in total, LDL, and HDL cholesterol levels, respectively, whereas only 31%, 19%, and 6% of neutral variants correlated with the respective lipid traits. Although putatively deleterious *APO* variants significantly correlated with cholesterol levels, no significant correlations were observed for serum triglyceride levels (*P* = 0.48; Fig. 4D). Notably, variants with the largest effect sizes on cholesterol traits were observed in *APOE* and *APOB,* whereas variants in *APOA4* and *APOA5* had the highest effects on triglyceride levels.

Because of the intimate link between lipid traits and CVD, we utilized the CARDIoGRAM GWAS data of 22,233 individuals with CAD and 64,762 controls (32) to further assess whether identified *APO* variants directly associated with disease risk. Sixteen missense apolipoprotein variants were covered by the CARDIoGRAM data, of which eight each were predicted to be deleterious or neutral (supplemental Table S6). Notably, whereas three of the putatively deleterious variants nominally correlated with CAD risk (*P* < 0.05; 38%), none of the correlations were found to be significant for the putatively neutral variants (0%). Combined, these data provide proof of concept that computational functionality predictions of apolipoprotein variants can highlight variants with putative effects on blood lipid traits and associated disease risk. We therefore conclude that the integration of NGS-based sequencing methods and in silico variant assessment provides a useful approach for the interpretation of the vast extent of rare or novel variants identified by large-scale sequencing projects for which clinical validations or GWAS data are not available.

## DISCUSSION

Serum lipid levels and risk of CVD are highly heritable with estimates ranging from 30% to 60% (56–58), and genetic polymorphisms in apolipoprotein-encoding genes constitute important modulators of serum lipid profiles and CVD susceptibility. Here, we analyzed the worldwide frequencies of 38 human *APO* alleles that have been consistently implicated in lipid traits and disease risk (Table 1). Most alleles exhibited large interethnic differences in frequencies, indicating that accurate genetic prediction of dyslipidemia and CVD risk requires population-specific genotyping strategies.

Importantly, common variants identified in large-scale GWASs only explain around 10–20% of the heritability of lipid traits (31, 39, 59). Rare SNVs are enriched in variants with functional effects and large effect sizes (60, 61) and have been suggested as an important source of this unexplained heritability (62, 63). However, the extent to which rare variants contribute to the overall functional variability in apolipoproteins had not been assessed. Thus, we leveraged population-scale NGS data to directly estimate the relative importance of rare genetic variability for the missing heritability of apolipoprotein-associated disease traits. By integrating the results of five partly orthogonal methods, we predict that for *APOE*, *APOB*, *APOH*, and *APOA5*, common variants explain 50–90% of the genetically encoded functional variability in coding sequences. In contrast, no common deleterious variant was detected in *APOC2* and *APOC3*. Thus, rare variants and CNVs are expected to explain the entire functional variability of *APOC3*, a gene for which loss-of-function mutations have been strongly linked with favorable lipid profiles (64), and of *APOC2*, which has been consistently associated with hypertriglyceridemia (65). These findings align with the causal implication of a multitude of rare variants in these genes with lipid traits, whereas no common deleterious variants in the coding sequences have been described.

Although the presented analyses provide the most comprehensive overview of genetic variability in *APO* genes described to date, it is important to note that variant and allele frequencies can differ drastically between ethnic groups within these aggregated superpopulations (66). Furthermore, genetic profiles of populations not represented in this data set might yield exciting additional information about apolipoprotein diversity. Examples for such insights based on population isolates or founder populations are effects of the triglyceride-lowering *APOC3* variants rs147210663 in Pima Indians (MAF = 2.6%) (48) and rs138326449 in Hutterites (MAF = 2.2%) (67), as well as the LDL risk variant rs5742904 in *APOB* in Old Order Amish (MAF = 12%) (68). With decreasing sequencing costs, we anticipate that the sequencing of founder populations will continue to represent a powerful tool for genetic research of apolipoproteins.

We used in silico prediction algorithms that can distinguish deleterious from functionally neutral missense variants with relatively high confidence as judged by areas under the receiver operating characteristic curve between 0.8 and 0.95 for genome-wide analyses (supplemental Table S2). However, for families of genes with low evolutionary pressure, the quality of predictions can be substantially lower (69). Importantly, we show that functionality scores for apolipoprotein variants were overall predictive for their effects on serum cholesterol levels despite complex linkage disequilibria, which might obscure functional effects (supplemental Fig. S2). For instance, rs1367177 and rs679899 were both predicted to affect APOB functionality and associate clearly with LDL (*p*_LD_ = 9 × 10^−183^ and 4 × 10^−39^, respectively) and total cholesterol levels (*p*_TC_ = 2 × 10^−139^ and 4 × 10^−22^, respectively). However, these variants are in linkage with the putatively neutral variant rs1801701 (*R*^2^ in Europeans = 0.18), which also correlated with LDL and cholesterol levels, albeit less strongly (*p*_LDL_ = 8 × 10^−15^ and *p*_TC_ = 2 × 10^−21^).

Although this predictive power is sufficient to provide faithful estimates of the overall functional mutational burden in the coding sequence of loci of interest on a population scale, these functional analyses are currently limited to individuals of European descent, as functional GWAS data for other populations of similar extent are currently lacking. Moreover, extensions of computational algorithms are needed to further improve the prediction of personalized dyslipidemia risks. Potential refinements include the consideration of population-specific linkage information, as well as adjustments that allow the functional interpretation of regulatory variants in promoters, enhancers, or UTRs that cannot currently be accurately evaluated using most computational methods. Finally, the evaluation of expression quantitative trait loci in relevant tissues, such as liver and small intestine, as provided by the Genotype-Tissue Expression Consortium, might allow further mechanistic interpretations of genetic apolipoprotein variation (70).

In summary, our analyses reveal that the genetic landscape in human apolipoproteins is highly complex, and every individual was found to harbor on average 19 *APO* variants, of which 2 had putative functional effects. The vast majority of variants were rare, and these rare variants contributed substantially to the genetically encoded apolipoprotein variability. Furthermore, by leveraging GWAS data from the CARDIoGRAM and Global Lipids Genetics consortia, we found that computational methods provide overall useful predictions for the functional effects of apolipoprotein variants on lipid traits and apolipoprotein-associated disease risk.

## Supplementary Material

Supplemental Data
