# Supplementary material for: Global genetic diversity of human apolipoproteins and effects on cardiovascular disease risk
Source: J Lipid Res. 2018 Aug 3;59(10):1987–2000. doi: 10.1194/jlr.P086710 (PMC6168301; doi:10.1194/jlr.P086710)
Supplement: Supplemental Data [file supp_59_10_1987__index.html]

Global genetic diversity of human apolipoproteins and effects on cardiovascular disease risk — Global genetic diversity of human apolipoproteins and effects on cardiovascular disease risk — Supplemental Data 

# Global genetic diversity of human apolipoproteins and effects on cardiovascular disease risk

## Supplemental Data

- Supplemental Table 3 (.xlsx, 46 KB) - Evolutionary constraint metrics of human APO genes.
- Supplemental Table 6 (.xlsx, 87 KB) - List of APO variants with available genotype association data from the Global Lipids Genetics and CARDIoGRAM consortia.
- Supplemental Table 5 (.xlsx, 55 KB) - Frequency information of APOB mutations related to hypobetalipoproteinemia.
- Supplementary Table 4 (.xlsx, 960 KB) - Overview of all putatively functional indels, single nucleotide variants and copy number variations in human APO variants.
- Supplemental Figure 1 (.pdf, 364 KB) - The fraction of rare functional variability that is allotted to rare variants differs considerably between genes and populations.
- Supplemental Figure 2 (.pdf, 781 KB) - Linkage overview of variants with available lipid trait association data provided by the Global Lipid Genetics Consortium.
- Supplemental Table 1 (.xlsx, 41 KB) - Complexity of analyzed human APO genes.
- Supplementary Table 2 (.docx, 99 KB) - Overview of functionality prediction methods used in this study.
