# Supplementary material for: Global genetic diversity of human apolipoproteins and effects on cardiovascular disease risk
Source: J Lipid Res. 2018 Aug 3;59(10):1987–2000. doi: 10.1194/jlr.P086710 (PMC6168301; doi:10.1194/jlr.P086710)
Supplement: Supplemental Data [file 10.1194_P086710_jlr.P086710-6.pdf]

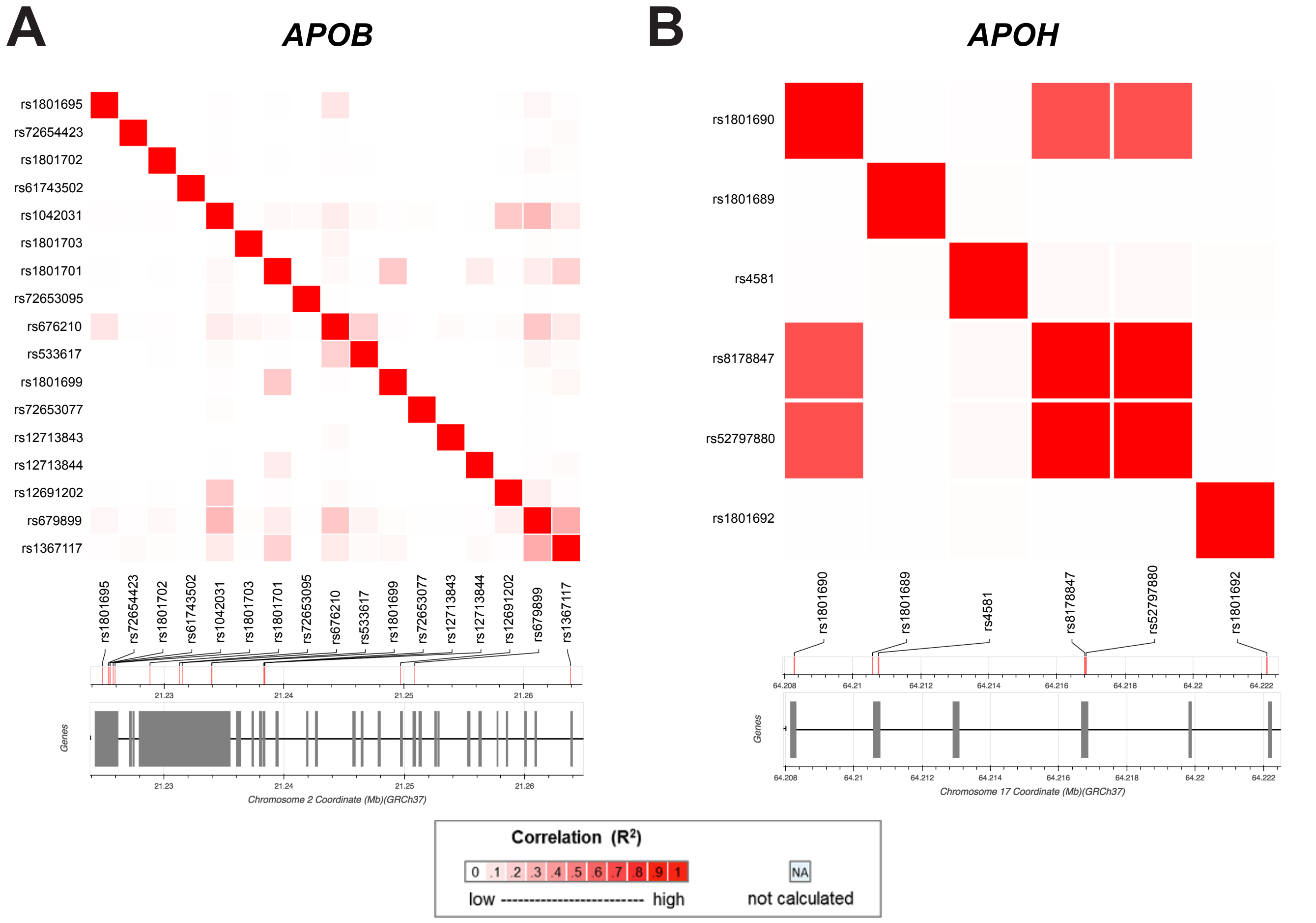

Supplementary Figure 2: Linkage overview of variants with available lipid trait association data provided by the Global Lipid Genetics Consortium. Linkage disequilibria for variants in *APOB* (**A**) and *APOH* (**B**) are shown.
