# Supplementary material for: Global genetic diversity of human apolipoproteins and effects on cardiovascular disease risk
Source: J Lipid Res. 2018 Aug 3;59(10):1987–2000. doi: 10.1194/jlr.P086710 (PMC6168301; doi:10.1194/jlr.P086710)
Supplement: Supplemental Data [file 10.1194_P086710_jlr.P086710-8.docx]

**Supplementary Table 2:** **Overview of functionality prediction methods used in this study.** AUC_ROC_ = area under the receiver operating characteristic curve.

| **Method** | **Category** | **Basis of decision** | **Threshold** | **Predictive performance (AUC_ROC_)** | **Reference** |
| --- | --- | --- | --- | --- | --- |
| SIFT | Functionality prediction algorithms | Support vector machine utilizing sequence conservation metrics that make use of Dirichlet priors. | <0.05 | 0.76 – 0.88 | ^1^ |
| PolyPhen-2 |  | Naïve Bayes method considering multiple sequence alignment scores, accessible surface area, hydrophobic propensities and B-factors. | >0.447 | 0.79 – 0.88 | ^2^ |
| Mutation Assessor |  | Evolutionary conservation patterns within protein families and across species using combinatorial entropy. | >1.9 | 0.8 – 0.83 | ^3^ |
| PROVEAN |  | Alignment-based score that can also assess in-frame insertions, deletions, and multiple amino acid substitutions. | <-2.5 | 0.85 | ^4^ |
| DANN | Ensemble score | Deep neural networks derived integrated score considering conservation metrics, regulatory information, protein or amino acid scores | >0.99 | 0.95 | ^5^ |

**Associated references:**

1. Ng PC, Henikoff S. Predicting deleterious amino acid substitutions. *Genome Research*. 2001;11:863-874

2. Adzhubei IA, Schmidt S, Peshkin L, Ramensky VE, Gerasimova A, Bork P, Kondrashov AS, Sunyaev SR. A method and server for predicting damaging missense mutations. *Nature Methods*. 2010;7:248-249

3. Reva B, Antipin Y, Sander C. Predicting the functional impact of protein mutations: Application to cancer genomics. *Nucleic Acids Research*. 2011;39:e118-e118

4. Choi Y, Sims GE, Murphy S, Miller JR, Chan AP. Predicting the functional effect of amino acid substitutions and indels. *PLoS ONE*. 2012;7:e46688-46613

5. Quang D, Chen Y, Xie X. Dann: A deep learning approach for annotating the pathogenicity of genetic variants. *Bioinformatics*. 2015;31:761-763
